# Public Health System Perspective on Implementation of Evidence-Based Fall Prevention Strategies For Older Adults

**DOI:** 10.3389/fpubh.2014.00191

**Published:** 2015-04-27

**Authors:** Sallie R. Thoreson, Lisa M. Shields, David W. Dowler, Michael J. Bauer

**Affiliations:** ^1^Colorado Department of Public Health and Environment, Denver, CO, USA; ^2^Oregon Health Authority, Portland, OR, USA; ^3^Multnomah County Health Department, Portland, OR, USA; ^4^New York State Department of Health, Albany, NY, USA

**Keywords:** state health departments, evidence-based strategy, older adults, fall prevention, health promotion

## State Health Departments’ Mission and Strategies

State health departments have traditionally worked in many areas of public health, including injury prevention (1). The public health approach toward injury and disease prevention directs programs to examine surveillance data and then design, implement, and evaluate strategies to address problems, such as falls among older adults (1, 2). The emphasis is to select evidence-based strategies that have been successfully tested in research settings and translated into programs that are readily available for implementation. Fall prevention among older adults has been acknowledged as a priority topic area, and one for which evidence-based strategies have been identified (1, 3).

## State Role in Fall Prevention

In 2011, the Centers for Disease Control and Prevention (CDC) funded state health departments in New York, Colorado, and Oregon to implement evidence-based older adult fall prevention programs over a 5-year period in several communities. The grantees were tasked to bring or expand the evidence-based programs of Stepping On, Tai Chi: Moving for Better Balance and the Otago Exercise Program to community-dwelling older adults (4). In addition, the grantees promoted the clinic-based stopping elderly accidents, deaths, and injuries (STEADI) toolkit developed by the CDC to improve medical providers’ falls prevention assessment and treatment, emphasizing referrals to the evidence-based programs in their communities (5). Each of the three states decided that instead of directly providing programing by state-level agencies, they would partner with local organizations to build infrastructure, change policies, and increase delivery and sustainability of the evidence-based programs. This commentary shares the experience from our three states after 2.5 years of efforts to build clinical and community prevention efforts to reduce falls in older adults.

## Successes

The goal of the state health departments was to go beyond “business as usual.” The states worked to develop innovative partnerships to effectively reach target audiences. As illustrated with specific examples in Table 1, successful implementation of the programs by each state health department can be attributed to a number of factors. First, each state ensured that internal support for the program was integrated within the structure and function of the state health department. Second, the states disseminated the programs through a variety of creative partnerships with health care and community-based organizations not traditionally involved with public health. Third, the states learned to understand and work with the needs of their partner organizations. An important lesson for working with health care partnerships was to acknowledge their business goals and consider initiatives meaningful to each organization. Next, the state health departments made it a priority to assist the local partners with embedding the evidence-based programs within their organizational structure. This entailed building a state infrastructure for instructor training, helping partners see and develop their roles in falls prevention, and providing the technical expertise to share marketing strategies so partners could ensure their programs effectively reach the older adult audience. Lastly, each state health department applied evaluation techniques to provide feedback to the partners on the positive outcomes of the programs, and to initiate program changes when a strategy was not working.

**Table 1:** **Factors to successful implementation of a fall prevention program for older adults**.

Examples of specific strategies employed by three state health departments (CO, OR, and NY) in implementing a fall prevention program for older adults	
Building public health infrastructure	State Health Department strategic plan includes a section recommending evidence-based programs for fall prevention for older adults
	Injury Community Planning Group includes falls prevention as a priority topic
	Public health toolkits for Accountable Care Organizations and Patient Centered Medical Homes include recommendations for falls prevention interventions to meet quality standards and clinical incentive measures
Developing new partners	Formed relationships with new partners, e.g., specific fitness centers, local parks and recreation departments, community health workers, YMCAs, and home health agencies
	Engaged with physician practice groups, professional associations, and health insurance companies to reach health systems and individual physician practices
	Worked with state-level professional organizations such as physical therapy association, primary care association, pharmacy association, and state parks and recreation association to encourage their joint role in fall prevention
Developing capacity for technical assistance	For the Otago program, the University of North Carolina developed a web-based training for physical therapists and an on-line database to track Otago patients
	Health department staff developed expertise on EHRs and the use of health care transformation initiatives to develop system-wide improvements in health care
	CDC developed a system to provide physicians with Maintenance of Certification and Continuing Medical Education credits for participation in the STEADI program
Facilitating program uptake in organizations	Developed state-wide training systems to certify Stepping On and TCMBB instructors
	Many local parks and recreation departments added TCMBB to their regular class schedule
	Developed physician and physical therapy champions who led their clinic teams in successfully implementing STEADI at the practice level
Facilitating program uptake in systems	Medicare-beneficiary fitness programs (Silver Sneakers and Silver and Fit) added TCMBB to their approved program list in one state
	Stepping On was adopted by hospital systems as a key injury prevention program for clinics and trauma centers
	Stepping On was added as standard program by a Veterans Administration Medical Center
Reaching underserved populations	Spanish-speaking health promoters and parish nurses were trained to deliver classes
	Spanish language version of Stepping On is under development
	Classes were offered at churches and senior residential housing complexes in addition to clinics and fitness centers
	Small program subsidies were used to reach underserved seniors who are minorities, non-English speaking, or disabled
Evaluate programs for fidelity and success	Data collection tools were developed to track programs
	Clear and open communication with partners was established

## Challenges Moving Forward

All of the states developed program implementation strategies to meet these challenges:

- It took substantial time and effort to embed these programs into existing infrastructure within the state health departments and their partners. The comprehensive integrated approach requiring simultaneous implementation of four programs was a definite challenge.
- Recruiting and implementing STEADI with health care providers was difficult. Health care entities were reluctant to partner with public health agencies given the demands of the clinic practice and multiple initiatives already being promoted. Part of the challenge was the need for rapid education of health department staff in electronic medical records and Medicare billing and coding. Additionally, identifying and motivating champions within medical practices and physical therapy agencies to lead the process was problematic. In particular, medical practices do not have a strong history of patient referral to community-based programs, despite the value of those programs being well documented in the medical and public health literature (6–8).
- Implementing a clinical intervention such as Otago was challenging due to Medicare billing requirements as well as the lack of Otago experts in any of the funded state health departments. The web-based Otago training was essential for training physical therapists, although it is still uncertain exactly how the program is being implemented with patients.
- The states developed relationships with specific partners in order to ensure sustainable programing for vulnerable and underserved elderly, such as minorities, non-English speakers, and those with disabilities.
- Evidence-based programing and the need to maintain essential elements of adoption and fidelity were new concepts to many community partners. This is an area where state health departments provided technical assistance and direction (9).

## Conclusion

The three states have demonstrated success in implementing evidence-based programing for fall prevention among older adults at the community level. Implementation of strategies to not only sustain but also to increase activities to penetrate the much larger state-wide older adult community remains challenging. The key to success will be to recognize fall prevention activities as an essential service in patient care and health promotion for older adults. The state health departments will continue to engage with community partners willing to make commitments to integrate fall prevention into their regular activities and to identify sustainable sources of funding and reimbursement to maintain these programs (8).

## Conflict of Interest Statement

The authors declare that the research was conducted in the absence of any commercial or financial relationships that could be construed as a potential conflict of interest.

*This paper is included in the Research Topic, “Evidence-Based Programming for Older Adults.” This Research Topic received partial funding from multiple government and private organizations/agencies; however, the views, findings, and conclusions in these articles are those of the authors and do not necessarily represent the official position of these organizations/agencies. All papers published in the Research Topic received peer review from members of the Frontiers in Public Health (Public Health Education and Promotion section) panel of Review Editors. Because this Research Topic represents work closely associated with a nationwide evidence-based movement in the US, many of the authors and/or Review Editors may have worked together previously in some fashion. Review Editors were purposively selected based on their expertise with evaluation and/or evidence-based programming for older adults. Review Editors were independent of named authors on any given article published in this volume*.
